# Efficacy of tamsulosin versus tadalafil as medical expulsive therapy on stone expulsion in patients with distal ureteral stones: A randomized double-blind clinical trial

**DOI:** 10.1590/S1677-5538.IBJU.2020.1007

**Published:** 2021-05-20

**Authors:** Siavash Falahatkar, Ardalan Akhavan, Samaneh Esmaeili, Atiyeh Amin, Ehsan Kazemnezhad, Alireza Jafari

**Affiliations:** 1 Guilan University of Medical Sciences Razi Hospital Urology Research Center Rasht Iran Urology Research Center, Razi Hospital, School of Medicine, Guilan University of Medical Sciences, Rasht, Iran

**Keywords:** Ureteral Calculi, Tamsulosin, Tadalafil

## Abstract

**Purpose:**

To compare the effects of tadalafil, tamsulosin, and placebo as a medical expulsive therapy (MET) for distal ureteral calculi.

**Materials and Methods:**

This prospective randomized double-blind clinical trial was conducted on 132 renal colic patients with distal ureteric stones (≤10mm) over a period of 12 months. Patients were randomly divided into three groups. Patients in group A received tamsulosin 0.4mg, in group B received tadalafil 10mg, and in group C received placebo. Therapy was given for a maximum of 4 weeks. The rate of stone expulsion, duration of stone expulsion, the dose and the duration of nonsteroidal anti-inflammatory drugs (NSAIDs), analgesic use, and adverse effects of drugs were recorded.

**Results:**

Demographic profiles were comparable between the 3 groups. Although the stone expulsion rate in group A (72.7%) was higher in comparison to group B(63.6%) and group C(56.8%), it was not considered statistically significant (P=0.294). Shorter mean time to stone expulsion was significantly observed in group A (17.75±75), than group B(21.13±1.17) and group C(22.25±1.18) (P=0.47). The mean number of analgesic use was 9.8±5.09 days in group A, 14.6±7.9 days in group B, and 12.6±22.25 days in group C, this difference was significant (P=0.004). The analgesic requirement (doses of NSAIDs and pethidine) in group A was significantly lower than other groups (P<0.05). Also, patients in group A reported fewer headaches compared to other groups (P=0.011).

**Conclusion:**

Tamsulosin as medical expulsive therapy is more effective for distal ureteric stones with less need for analgesics and less stone expulsion time than tadalafil.

## INTRODUCTION

Nephrolithiasis is one of the most commonly diagnosed urologic diseases with a rising prevalence, with great economic and clinical burden on the health care system (1). Studies reported different incidence rate of nephrolithiasis and it varies in different population around 12% in adult men and up to 6% in adult women. The prevalence of nephrolithiasis reaches its peak in population aged 20-40 years. The probability of a urinary stone varies according to several factors such as age, sex, race and geographical area (2, 3).

Twenty-two percent of nephrolithiasis are ureteral stones and 68% of ureteral stones are found in the distal part (2). The clinical presentation of stones mainly includes colic pain and urinary symptoms such as urinary frequency (4). A number of factors are involved in determining the treatment of ureteric stones. These factors are divided into four broad categories including stone factors, clinical factors, anatomic factors and technical factors. In many cases, based on the patient's preference and consideration in achieving higher stone-free and lower side effects of the procedure, more than one treatment method is appropriate (2, 5–7).

The current curative options for ureteral stones range from medical treatment to surgical interventions. The rate of stone passage in the distal ureter is reported 75% based on a meta-analysis (8). According to American Urological Association's (AUA) guideline, stones smaller than 5mm have a 68% chance of passing while it decreases to 47% for larger stones (6-10mm).

For large proximal ureteral stone ≥10mm various surgical options such as extracorporeal shock wave lithotripsy (ESWL), ureteroscopic lithotripsy (URSL), laparoscopic ureterolithotomy (LU) and percutaneous nephrolithotomy (PCNL) are suggested in many studies (9, 10).

Medical expulsive therapy (MET) is an approved method to increase the chance of stone passage in both American and European Guidelines. MET contains various drugs such as alpha adrenoreceptor antagonists, calcium channel blockers and prostaglandin inhibitors. Phosphodiesteras e type 5 inhibitors (PDE5-Is) were more recently approved in the treatment of urinary tract symptoms (1, 11). However, the most commonly used drugs in MET are still alpha-blockers, among which tamsulosin is more popular. The probable mechanism of action of tamsulosin as a MET is the selective relaxation of ureteral smooth muscle (12). It appears that in the smooth muscles of the ureter, especially in the distal one-third, alpha receptor is also expressed, and the specific blockage by tamsulosin leads to muscle relaxation, increasing the chance of stone passage, reducing the time of expulsion. Several studies have advocated the use of tamsulosin in stone passage. Although positive evidence exists in favor of stone passage by tamsulosin, meta-analysis (12, 13) and a large multicenter, randomized, placebo-controlled trial by Pickard (14) have not proven these positive effects.

On the other hand, tadalafil (a PDE5-Is) has been also suggested many studies in the treatment of lower urinary tract symptoms (LUTS) secondary to benign prostatic hyperplasia (BPH) in recent years. Tadalafil causes the prostate smooth muscle relaxation via the nitric oxide (NO)-cyclic guanosine 3’, 5’-monophosphate (cGMP) pathway and thereupon improves LUTS and the function of the cavernous muscles in cavernous artery. In recent studies, the administration of PDE5-Is alone and in combination with tamsulosin has led to acceleration of stone passage or even reduction of stone expulsion time and need for analgesics (11).

Since the reported results of the studies cannot conclusively answer the question of whether the rate and time of stone expulsion and analgesic requirement time are the same among patients treated with tamsulosin, and tadalafil or not, in this study, we aimed to evaluate the results of using tamsulosin, which is currently controversial in passing renal stone (13, 15) with tadalafil, among patients with distal ureteral stone.

## MATERIALS AND METHODS

In this double-blind randomized clinical trial, between November 2017 to November 2018, 132 patients with lower ureteral stones referred to the urology clinic of Razi Educational Hospital were studied. According to the random block method, six blocks were produced for 132 patients. Then, each patient was assigned a specific code. Neither the patient nor the treating physician were aware of which type of medication was given. The study was conducted in accordance with the Declaration of Helsinki. It was approved by the Ethics Committee of Guilan University of Medical Sciences (IR.GUMS.REC.1396.41) and it was also registered online at Iranian Registry of Clinical Trials (http://www.irct.ir//:IRCT201709191853N14). The informed written consent was signed and dated by all participants before participating.

Adult aged 18-64 years who suffered from renal colic and single distal ureteral stone smaller than 10mm were included in the study. Diagnosis of colic and distal ureteral stones were performed by ultrasound or computerized tomography (CT) scan without intravenous contrast. In the current study, distal ureter was defined below the bifurcation of Iliac vessels.

Patient with fever more than 37.8°C, GFR ≤30, single kidney, multiple ureteric stones, history of ureteral surgery, diabetes, gastric ulcer, usage of alpha-blocker drugs, calcium channel blocker and nitrate, pregnancy or any kind of allergy to the drugs were not included. Patients in need of surgical or endoscopic intervention and patients with acute and resistance renal colic pain, uremic symptoms, urinary retention, and patients who wanted urgent medical intervention were excluded. The acute and resistance renal colic was defined as the pain which cannot be controlled by a standard dose of analgesics (100mg diclofenac and 50mg pethidine) (2).

For each patient admitted to the study, a checklist was filled out. The medical history of all participants and the result of their physical examination were recorded. Also, patient's blood urea nitrogen (BUN) and serum creatinine levels were measured. Due to a significant financial burden for patients, CT scan was not done in all subjects. CT scans without contrast were performed for patients with renal colic pain and urinary stone symptoms just in case of not seeing stones on their ultrasound.

Based on a power of 80% with 95% confidence interval and using the results of the study of Puvvada et al. (2), a sample size of 44 patients in each group was needed to determine the expected clinical difference of 25%.

One hundred and thirty-two patients were randomly allocated to three groups (A, B and C). The patients in group A received tamsulosin 0.4mg (Farabi Medicine Pharmacy, Iran) once daily, in group B received tadalafil 10mg (Razak Medicine Pharmacy, Iran) once daily, and those in control group (group C) were given the placebo treatment once daily. Medication continued to be taken until stone expulsion or up to 4 weeks. Participants were asked to report any symptoms or complications during this period.

Patients were advised to pass their urine in a filter or similar. They were also asked to report the time of stone pass when they observed stones in the filter.

Expulsion of stone was confirmed with a CT scan without contrast at the end of the 4th week. In case of seeing stone in the CT scan image, patients received immediate endoscopic intervention; otherwise, stones were supposed of having passed.

All drugs (tamsulosin, tadalafil, and placebo) were identical in shape, size, and color. The drugs were packaged in same three distinct boxes (A, B, C) by a project associate (other than the principal researcher and analyst). Each of the drug packages was selected for the patients based on six randomized blocks.

Data were entered into SPSS Version 23 and the comparison of the frequency of variables was analyzed by the Chi-Square test and by variance analysis using the Post hoc Tukey test. Regression methods were used to determine the therapeutic effects of the interventional variables compared to the placebo. The level of significance P-value was less than 0.05.

## RESULTS

All 132 patients completed the treatment and follow-up period. Demographic data of patients are given in Table-1 for all three groups. CT scan was performed in 95 (71.96%) patients for diagnosing of ureteral stone. The mean age of the patients was 37±11.35 years in group A, 37.36±12 in group B years, and 36.9±11.53 years in group C. According to results, 53.8% of patients were male, and 46.2% were female. The results showed that there was no statistically significant difference between age, sex, BMI and the size of the stone in A, B and, C groups (Table-1). The frequency of expulsed stone in group A was 72.7%, group B was 63.6%, and group C was 56.8%. There was no significant difference in expulsed stone between A, B and, C groups (p=0.294).

**Table 1:** Demographic Characteristics and Clinical Outcomes in the 3 groups.

Groups	(Group A) Tamsulosin	(Group B) Tadalafil	(Group C) Placebo	P-value
Male/Female, n	24/20	23/21	24/20	0.97*
Age, years (Mean±SD)	37±11.35	37.36±12	36.9±11.53	0.981**
BMI, kg/m^2^ (Mean±SD)	26.78±1.85	26.52±1.92	26.13±1.95	0.286**
Stone size, mm (Mean±SD)	6.93±1.46	6.86±1.65	6.88±1.48	0.978*
Frequency of expulsion of stone, n (%)	32 (72.7)	28 (63.6)	25 (56.8)	0.294*
Expulsion of stone time, days (Mean±SD)	17.75±75	21.13±1.17	22.25±1.18	0.046***
Doses of used NSAID, mg (Mean±SD)	818.18±618.05	1068.2±503.3	1095±503.3	0.038**
Doses of used Pethidine, mg (Mean±SD)	165.9±219.6	270.45±170.9	254.54±54	0.04**
Mean analgesic requirement time, days (Mean±SD)	9.8±5.09	14.6±7.9	12.6±22.25	0.004**
Side effects, n (%)	14 (31.8)	14 (31.8)	2 (4.5)	0.002*

* Chi square test

** One Way ANOVA test

*** Tarone - Ware test

The mean time of stone expulsion in the group A was 17.75±75 days, while it was 21.13±1.17 days in group B, and 22.25±1.18 days in the group C, which it seems tamsulosin had a better effect on stone expulsion than tadalafil, but these differences did not reach the level of significance (p=0.46) (Table-1).

Additionally, the mean dose of used NSAIDs in group A was 818.18±618.05mg, in group B was 1068.02±503.3mg, and in the group C was 1095±503.3mg. It is interesting to be aware that the patients who used tamsulosin had significantly less need for analgesics than other groups (p=0.038) but there was no notable difference between tadalafil and placebo. We found that the patients who used tamsulosin needed significantly less pethidine than other groups, too (p=0.04) (Table-2). There were no significant differences in the frequency of expulsion of stone (p=0.294) and the frequency of endoscopic treatment (p=0.294) between the three groups (Table-1).

In terms of drug-related adverse, including headache, dizziness, orthostatic hypotension, back pain, and retrograde ejaculation, there was just a significant difference in headache between the three groups. Seven patients (15.9%) in tadalafil group reported headaches during the study, which was significantly higher than number of reported headaches in tamsulosin 2 (4.5%) and the placebo group 0 (0.0%) (p=0.011). There was no complication among the patients of group C. Although orthostatic hypotension and retrograde ejaculation reported in 2 (4.5%) and 3 (6.8%) cases of group A, respectively, those in group B experienced none of them (p=0.106). Out of 5 patients who had back pain, 1 (2.3%) case was in group A and 4 (9.1) were in group B (p=0.126). Dizziness was also reported in 7 cases (5 (11.4%) in group A and 2 (4.5%) in group B) (p=0.069).

At the end of the study period, endoscopic interventions were suggested for those who did not passed the stone by MET (in abdominopelvic CT scan) in group A, B and C [12 (27.3%), 16 (36.4%) and 18 (43.2%), respectively], however the difference was not considered statistically significant (p=0.294).

## DISCUSSION

Distal ureteral stones are the most symptomatic calculi. Studies reported an overall spontaneous passage rate of 25% to 51% for distal urethral stones sized 5 to 10mm and 71% to 98% for stones smaller than 5mm (16–19).

Considering the effect of MET on the reduction of symptoms and facilitation of stone expulsion, it is highly recommended treatment modality to increase stone expulsion rat (2, 20, 21). Alpha-blockers, calcium channel blockers (CCB), and PDE5-Is in MET have been used to improve stone passage and decrease the need for analgesics (17). The role of adjunctive MET with tamsulosin on ureteral stone expulsion has been reported (22).

Although many studies reported that tadalafil is more effective than tamsulosin in facilitating stone expulsion (20–23), in the current study, the rate of stone expulsion in the tamsulosin group was higher than tadalafil and placebo groups (72.7%, 63.6% and 56.8%, respectively). However, this difference did not reach statistical significance (P=0.294). In the study of Al-Hossona et al. (23), tadalafil significantly improved stone expulsion in comparison with placebo. Also, many studies and meta-analysis showed that tamsulosin combined with tadalafil was associated with significantly higher stone expulsion rate compared with tamsulosin alone (21, 24, 25),

Comparing the time of stone expulsion between groups in our study, we found in tadalafil group that patients had lower time than placebo groups but not the tamsulosin group (21.13±1.17 vs. 22.25±1.18 and 17.75±75, respectively). Patients in tamsolusin group had significantly lower expulsion time than other two groups (p=0.046) Our results however do not match with those of older studies (2, 20) which concluded tadalafil has a significantly higher stone expulsion time than tamsulosin. Our study also demonstrated that tadalafil was not better than placebo in accelerating stone expulsion in contrast to Al-Hossona et al. (23).

Even meta-analyzes have reported conflicting results. While a meta-analysis by Li et al. (24) showed that the time to expel stones in tadalafil group was significantly less than tamsulosin group (p=0.028), another meta-analysis by Liu et al, (25) in the same year reported no significant shorter stone expulsion time for tadalafil in comparison with tamsulosin.

In the current study, tamsulosin had the ability to decrease the need for the analgesic (pethidine and/or NSAID), in comparison with tadalafil. The results showed that tadalafil not only did not reduce the need for analgesics but also caused more requirement of analgesics. Interestingly, the outcome of our study is exactly in contrast with the majority of previous studies (2, 4, 21, 23), which reported that tadalafil is able to reduce the need for analgesics. Jayant et al. (21) also reported that the mean number of times of analgesic use in tadalafil group was significantly lower than tamsulosin group (p=0.000).

However, in 2019, Li et al. (24) in a meta-analysis showed that dosage of analgesia used in tadalafil group was significantly higher than tamsulosin group and the duration of analgesia use in patients who used tamsulosin plus tadalafil were significantly lower than those who received tamsulosin alone.

The average used analgesic dose has been reported about 200mg, and was 130mg in Kumar, et al. (4) and Kc, et al. (20) studies, respectively. While the mean analgesic dose in our study was about 2-3 times more than their findings.

In our study, the reported side effects were mild to moderate, transient and well tolerated in all three groups, perhaps because our study population was young without any comorbidity. And that's why all the patients continued treatment until the end of the study.

The statistical significance of side effects in our study is due to the low rate of reported side effects in the placebo group and the occurrence of adverse effects was equal in the two groups of tadalafil and tamsulosin. Only the frequency of headache was significantly higher in the tadalafil group 7 (15.9%) than in the other two groups (p=0.011).

Therefore, we think an increase in the need for analgesics may have been due to the adverse effect caused by the use of tadalafil. Liu et al. (25), in their meta-analysis, showed that using tadalafil is associated with more side effects such as headache, dizziness, backache, and orthostatic hypotension than tamsulosin. But another meta-analysis by Li et al. (24), reported no statistic difference between tamsulosin group and tamsulosin plus tadalafil group in terms of drug's adverse effects (p >0.05).

Considering that headaches can cause various types of pain, an urologist will be more cautious about prescribing tadalafil as a MET. On the other hand, more use of analgesics, in this study, partially showed the low threshold of our patient's tolerance to pain, as well as the culture of a drug overuse in Iranian population.

Recently, however, many studies have been conducted to assess the effect of tadalafil on stone expulsion and have attracted the attention of urologists to use this drug as a MET, the findings of meta-analysis do not support complete replacement of tamsulosin with tadalafil and they only suggest that combination of tadalafil and tamsolusin in MET may reduce the need for SWL therapy and minimally invasive procedures (24, 25). Even in the EAU Guidelines 2020, the role of tadalafil in MET for distal ureteral stones has not been proved.

In addition, it seems that quite contrary to Pickard et al. (14), study, in which the role of tamsulosin in stone expulsion was somewhat questioned, this study defends the efficacy of tamsulosin in reducing pain, expediting expulsion and increasing expulsion speed.

## LIMITATIONS

Although our study had prospective randomization with the simultaneous presence of placebo, the findings had some limitations. This study was single-centered, therefore, the results require further investigation. Selection bias is able to limit the generalization of these findings because the type of participates in a city or a country could be different from the other cities or countries and could be related to descent diversities.

## CONCLUSION

Although both drugs are safe, effective, and well tolerated, the study has shown that tamsulosin is more efficacious than tadalafil as a medical expulsive therapy in reducing the stone expulsion time with better control of pain and less postoperative requirement for analgesic. So, single medical expulsive therapy by tamsulosin can be used safely for distal ureteral stone. However, further large, multicenter RCTs are needed to confirm these findings.
